# Sex Steroid Regulation of Oxidative Stress in Bone Cells: An In Vitro Study

**DOI:** 10.3390/ijerph182212168

**Published:** 2021-11-19

**Authors:** Valeria Sibilia, Daniele Bottai, Roberto Maggi, Francesca Pagani, Raffaella Chiaramonte, Domenica Giannandrea, Valentina Citro, Natalia Platonova, Lavinia Casati

**Affiliations:** 1 Department of Medical Biotechnology and Translational Medicine, Università degli Studi di Milano, 20129 Milano, Italy; valeria.sibilia@unimi.it (V.S.); francesca.pagani@unimi.it (F.P.); 2 Department of Health Sciences, Università degli Studi di Milano, 20142 Milano, Italy; daniele.bottai@unimi.it (D.B.); raffaella.chiaramonte@unimi.it (R.C.); domenica.giannandrea@unimi.it (D.G.); valentina.citro@unimi.it (V.C.); natalia.platonova@unimi.it (N.P.); 3 Department of Pharmaceutical Sciences, Università degli Studi di Milano, 20133 Milano, Italy; roberto.maggi@unimi.it

**Keywords:** bone, steroids milieu, histone modification, oxidative stress, H3K4me3, H4 acetylation

## Abstract

Environmental stimuli, including sex hormones and oxidative stress (OS), affect bone balance, modifying the epigenetic profiles of key osteogenic genes. Nonetheless, the interplay between sex steroids, epigenome and OS has yet be fully elucidated. This paper aims to study in vitro the role of sex steroids in OS-induced alteration in bone cells’ homeostasis, and to assess the possible contribution of epigenetic modifications. Toward this purpose, osteoblast (MC3T3-E1) and osteocyte (MLOY-4) cell lines were exposed to two different sources of free oxygen radicals, i.e., tert-butyl hydroperoxide and dexamethasone, and the protective effect of pre-treatment with androgens and estrogens was evaluated. In particular, we analyzed parameters that reflect bone cell homeostasis such as cell viability, cell migration, transcriptomic profile, transcriptional activity, and epigenetic signature. Our findings indicate that estrogens and androgens counteract OS effects. Using partially overlapping strategies, they reduce OS outcomes regarding cell viability, cell migration, the transcriptomic profile of gene families involved in bone remodeling, and epigenetic profile, i.e., H3K4me3 level. Additionally, we demonstrated that the protective effect of steroids against OS on bone homeostasis is partially mediated by the Akt pathway. Overall, these results suggest that the hormonal milieu may influence the mechanisms of age-related bone disease.

## 1. Introduction

The sexual steroid milieu (estrogens and androgens) is involved in acquiring bone mass during puberty and maintains this throughout adult life. A reduction in estrogen levels in females during menopause or estrogens and androgens in males later in life causes bone mass loss and lowers strength [1]. This unbalance contributes to the development of osteoporosis, one of the most common metabolic aging disorders [1]. The correct acquisition and maintenance of bone mass are essential for bone integrity and regeneration. Bone’s structural integrity is preserved by removing the old bone using osteoclasts (OC) and depositing new bone in place using osteoblasts (OB). Any factor that destroys the coupling between OB and OC action leads to a reduction in the quality and density of bone mass, resulting in decreased bone strength and increased fracture risk, as occurs in osteoporosis [2].

Estrogens (E2) and androgens are derived from cholesterol and synthesized in the gonads and the adrenals. During menopause, E2 levels abruptly decline; however, older men do not show true andropause, and their E2 concentration is at a sufficient level to maintain skeletal homeostasis [3,4,5]. As in other tissues, estrogen and androgen effects on bone are mediated by their binding with high affinity to ERs and the AR, respectively [1,6].

Notably, the roles of steroid receptors have been uncovered in the pathological progression of skeletal disorders, including estrogen-deficiency-induced osteoporosis in postmenopausal women [7]. The osteoprotective action of E2 is associated with a regulation of bone resorption and maintenance of normal bone remodeling [8] in both sexes; however, the specific inhibitor’s effect on osteoclastogenesis seems to be typical of females [9]. In osteoblasts, AR appears to play a direct anabolic function in the maintenance of bone metabolism [9], and the high strength of male bones is likely caused by the anabolic effects of androgens. AR functions are needed for the androgen-mediated inhibition of osteoclastogenesis [10].

Aging and estrogen deficiency are the two most critical factors in the development osteoporosis in both women and men. However, it is unknown whether the molecular changes caused by estrogen deficiency and aging are similar to, distinct from or influence each other. According to Manolagas, both female and male mice show all significant skeletal aging features independently of the lack of sex steroids [11], suggesting, for the first time, that the effects of aging and sex-steroid-deficiency on a mammalian skeleton are independent.

Steroid receptors are nuclear receptors and control the transcription of target genes. It is also known that steroid receptors interact with histone-modifying enzymes to regulate gene transcription and chromatin remodeling [12,13]. However, the direct target genes of sex-steroid action on bone remain poorly defined [1]. Steroid receptors can also mediate non-genomic effects (activating cytoplasmic kinases and downstream signaling by transcription factors such as Elk-1 and AP-1) or genomic effects, independently of their direct interaction with the steroid-responsive element on the DNA sequence [14]. Bone, in particular, seems to display ligand-independent effects, especially regarding ERα [15].

In recent years, several genes have been analyzed as putative targets of the antiresorptive effect of estrogens on the bone, from both cell lines and primary cell cultures. The putative target genes include interleukins (IL-1β, IL-6, IL-7), tumor necrosis factor (TNF)-α, M-CSF, RANK-L, and OPG [16]. Various genes in the osteoblast lineage cells have been proposed as targets of a putative “bone-forming” effect of estrogens, again based on evidence from in vitro studies with osteoblast-like cell lines. This list includes retinoblastoma-binding protein 1 (RBBP1, a RUNX coactivator), transforming growth factor (TGF)-β-inducible early gene-1 (TIEG, a modulator of OPG), GATA4, and alkaline phosphatase (ALPL) [17,18]. Over the years, experimental evidence has suggested several factors as mediators of E2 effects on osteoclasts [1].

Several pieces of evidence show that Erα, in osteoclasts, but not in osteoblasts, mediates the proapoptotic effect of estrogens [19]. Furthermore, estrogens have an antiapoptotic, not proapoptotic, effect on osteoblasts [8].

Wnt/β-catenin signaling is another critical pathway involved in estrogen action in bone. It was recently shown that Wnt/β-catenin signaling acts as a negative regulator of osteoclastogenesis [20]. It was demonstrated that mice with the deletion of β-catenin in osteoclast lineage cells exhibit an increased osteoclast number and decreased bone mass [21]. Additionally, Wnt ligands such as Wnt3a reduce osteoclast formation [21] and promote osteoclast apoptosis. Estrogens act on osteoclasts to increase the expression of β-catenin [22]. This finding may indicate that estrogens counteract bone resorption by directly potentiating Wnt signaling in osteoclasts.

In vitro evidence has suggested that androgens (DHT or T) directly act on osteoclast progenitors and mature osteoclasts to inhibit osteoclastogenesis and promote osteoclast apoptosis [8]. However, genetic evidence from mice with osteoclast-specific AR deletion indicates that androgen signaling in osteoclasts has no antiresorptive effect on the cancellous or cortical bone compartments. Indeed, mice with the targeted deletion of AR in osteoclast lineage cells exhibit no changes in osteoclast number or bone mass [23].

AR signaling in osteoblasts is responsible for the protective effects of male steroids on cancellous bone mass. This signaling leads to a decrease in osteoclast numbers and bone resorption. On the other hand, the effects of androgens on trabecular bone mass are partly mediated by late osteoblasts and osteocytes, although this remains unclear [1].

Mechanistic studies of the effects of sex steroid deficiency on the murine skeleton have revealed that a deficit of steroids (androgens and estrogens), similar to old age, leads to an increase in reactive oxygen species (ROS) in bone cells [24,25,26]. ROS source represents one of the mechanisms that favor bone aging [24]. On the other hand, the systemic administration of antioxidant compounds counteracts losses in bone mass due to sex steroid deficiencies in male and female mice [25,26]. These experiments have raised the possibility that an ROS increase may be a common mechanism of sex steroid deficiency and aging in bone homeostasis; moreover, sex steroid deficiency may accelerate the effects of aging on skeletal involution. Is it possible that epigenetics could be involved in this mechanism?

Several pieces of evidence support the idea that epigenetic modification plays a key role in aging and mediating ROS effects [27]. Epigenetic mechanisms play a relevant role in controlling bone remodeling [28] and are highly conserved in living organisms, starting from the yeasts [29] Epigenetic mechanisms are affected by environmental cues; some endocrine disruptors, such as PCBs, induce changes in chromatin state [12,30,31]. ROS represents one of these negative environmental cues. Senescence can be induced by inhibiting the enzyme histone deacetylase (HDAC), which promotes euchromatin formation [30]. Oxidative stress can influence the cell on multiple levels, from DNA and histones to histone modifiers, directly affecting the cellular epigenetic landscape [27].

The interplay between sex steroids, epigenetic mechanisms, and oxidative stress remains to be fully elucidated.

This paper aims to analyze in vitro the involvement of sex steroids in disruptions in bone metabolism associated with aging induced by free radicals and clarify the epigenome contribution.

MC3T3-E1 and MLOY-4 are used as pre-osteoblasts and differentiated osteoblasts and osteocytes, respectively.

MC3T3-E1 are characterized by distinct proliferation and differentiation stages; indeed, they reproduce a temporal program similar to osteoblast differentiation, as occurs during in vivo bone formation [31]. MLOY-4 cells [31], which are considered a suitable model to study osteocytes [32,33],were used.

We simulated the aging conditions that expose bone cells to both free radicals of oxygen sources, such as tert-butyl hydroperoxide (t-BHP) [34,35,36], and dexamethasone, an ROS source of glucocorticoid-induced osteoporosis [37]. To test the hormonal milieu effect, we pre-treated bone cells with androgens (DHT) and estrogen (17-β estradiol, E2), using a range of concentrations for the steroids that is as close as possible to the physiological/serological and pathological conditions (10^−9^ M–10^−12^ M) [38,39].

To understand the effect of steroids on aging bone cells induced by oxidative stress, we analyzed the parameters involved in bone homeostasis, such as cell viability and cell migration, and investigated the underlying mechanisms by studying the transcriptomic profile, transcriptional activity, and epigenetic signature.

## 2. Materials and Methods

### 2.1. Cell Culture

Murine osteoblastic cell line from ATCC (cat. CRL-2593), MC3T3-E1, were seeded in High-Glucose DMEM, (Euroclone, Milan Italy), supplemented with 10% FBS (Euroclone, Milan, Italy), as previously reported [34]. We replaced culture medium twice a week and MC3T3-E1 cells were trypsinized weekly. Murine osteocyte-like cells, MLO-Y4, were kindly provided by Dr. Romanello (Hospital “Santa Maria della Misericordia”, Udine, Italy). We coated plates of 10 mm with type I collagen. MLOY-4 cells were seeded as previously described. The cells were trypsinized twice a week.

To study MC3T3-E1 differentiation, cells were maintained as previously described [40]. In brief, cells were seeded in medium supplemented with l-ascorbic acid 50 μg/mL and β-glycerolphosphate (10 mM, both reagents from Merck KGaA (Darmstadt, Germany). Cultures were maintained for 7 days before experiments. All the experiments with steroids were performed in medium without phenol red and charcoaled FBS.

### 2.2. Cell Viability Assay

We analyzed the MC3T3-E1 and MLO-Y4 viability by seeding the cells at the density of 15 × 10^3^ cells/well in 96 multiwell plates. We analyzed the cell viability using MTT test, as previously reported [41].

### 2.3. Scratch Wound Healing Assay

A linear scratch on confluent MC3T3-E1 cells was performed by a sterile pipette. We removed the cellular debris by washing with PBS, and then we incubated the cells in DMEM 1% FBS alone or in the presence of various treatments for 24 h (see [41] for reference). We took the photographs immediately after the scratch (t0) and at various times after treatment (t18, t24, and t48) using an Olympus U-CMAD3 phase-contrast microscope equipped with a Zeiss Axiocam Icc1 camera at a 4× magnification. We analyzed the images using ImageJ software (NIH, Bethesda, MD, USA) as previously reported [42]. The covered area percentage was calculated for each experimental group by measuring the wound size at different times from treatment compared with the initial (t0) wound size considered as 100%.

### 2.4. Transcriptional Activity Analysis

Lipofectamine 2000 (ThermoFisher, Waltham, MA, USA) was used as a transfection agent and was added in 96-well plates according to the manufacturer’s protocol (see [12]). The following commercially available constructs (Promega, Milan, Italy) were transfected: pGL4.54 (luc2TK) used to normalize transfection efficiency (control reporter) and pNL (NlucP/TCF-LEF-RE) (experimental reporter) to analyze the β-catenin transcriptional activity that was revealed using the Nano-Glo Dual-Luciferase Reporter assay system (Promega) according to the manufacturer’s protocols. In brief, transfected MC3T3E1 cells were cultured in 96-well plates and treated for 48 h with E2 10^−9^ M or DHT 10^−9^ M and LiCl (20 mM) as a positive control (not reported in the figure) for β-catenin transcriptional activity. Each experiment was repeated three times.

### 2.5. Trascriptomic Profile Analysis

Adherent cells were harvested and total RNA was extracted using AURUM Column kit, according to the manufacturer’s instructions (Biorad). RNA pellet concentrations were spectrophotometrically assessed using microcuvette G1.0 in Eppendorf Biopohotometer.

For the PCR Prime Array, we used commercially available PCR arrays from SAB (Qiagen, Hilden, Germany) and Biorad (Hercules, CA, USA). We extracted the total mRNA using Biorad Mini kit, following the instructions. The RNA quality was evaluated with electrophoresis. The samples were read at the spectrophotometer. Total RNA (1 μg) was retrotranscribed using iScript Advanced cDNA kit from Biorad. For the PRIME PCR. We analyzed 10 μg of cDNA for well. We used both negative and positive controls for PCR, as requested by the assay. The reaction was carried out on QuantStudio Fast 5 (Thermofisher Scientific, Waltham, MA, USA), using 20 μL of the total volume for each well. Q-PCR was run following the manufacturer’s protocol (Biorad).

### 2.6. Western Blotting Analysis

Cells were collected in Laemmli buffer (62.5 mM Tris-HCl pH 6.8, 7.5% glycerol, 2% SDS, 0.125 M dithiotreitol), subjected to the Bioruptor (Diagenode, Liège, Belgium) for 5 min (30 s on; 30 s off, high power) prior to heating samples at 95 °C for 5 min. Protein extracts were then centrifuged at 13,200 rpm for 10 min and supernatants were saved and directly used for immunoblotting. Protein extracts were resolved by 15% polyacrylamide gel electrophoresis in the presence of SDS (15% SDS/PAGE) and transferred to 0.2-µpore nitrocellulose membranes by standard wet procedure [43]. After transfer, membranes were stained with Ponceau S to verify the correct transfer, and then blocked and probed overnight at 4 °C with the primary specific antibodies: anti-H3K4me3 (ref. Ab8580, Abcam, Cambridge, UK) and anti-H3 (ref. 1791, Abcam). Membranes were processed with WesternDot™ 625 Western Blot Kits (ThermoFisher, Waltham, Massachusetts, US), as described by the manufacturer. Fluorescent signals were quantified using ChemiDoc system (BioRad, Hercules, CA, USA). Data are expressed as mean ± SEM of the relative amounts of H3K4me3/H3 (arbitrary units).

### 2.7. Total HDACs I/II Enzymatic Activity

MC3T3E1 cells were seeded in 100 μL media per well in 96 wells at 1 × 105 cells/well and grown to 80% confluence. After the treatment (24 h 10−9 M steroids pre-treatment, and 3-h t-BHP treatment (250 μM or 125 μM), w followed the protocol from HDACs fluorimetric cellular activity assay kit (BML-AK503-0001 from Enzo Life Science). In brief, we added the substrate (Fluor de Lys™, Enzo Life Science, Milan, Italy) to each well for 4 h at 37 °C. Then, we added the developer for 15 min at 37 °C. We read the assay at 460 nm using the fluorimetric reader (Victor™, PerkinElmer, Waltham, MA, USA). We prepared a set of wells for Thricostatin 1 μM as internal control, as suggested by the assay.

### 2.8. H4 Global Acetylation Levels

According to the manufacturer’s protocol, histones were extracted from MC3T3-E1 using the Histone Extraction Kit (Abcam; #113476). Histone protein concentrations were measured using the BCA Protein Assay Kit (Pierce) (see [41] for details).

We detected the global histone acetylation level using the H4 Total Acetylation Detection Colorimetric Kit (ab115125), purchased from Abcam (see manufacturer’s protocol).

### 2.9. Statistical Analysis

We used GraphPad Prism8 software (GraphPad Software, San Diego, CA, USA) for all statistical analyses. The results are expressed as the mean ± SEM of at least 3 independent experiments (6 replicates for each experiment). Differences between groups were evaluated by one-way or two-way ANOVA, followed by Bonferroni post-hoc test, if appropriate. We considered a *p*-value less than 0.05 as significant.

## 3. Results

### 3.1. Effects of Steroids on Counteracting t-BHP-Induced Oxidative Stress in Pre-Osteoblast MC3T3-E1 Cells

**Figure 1:**
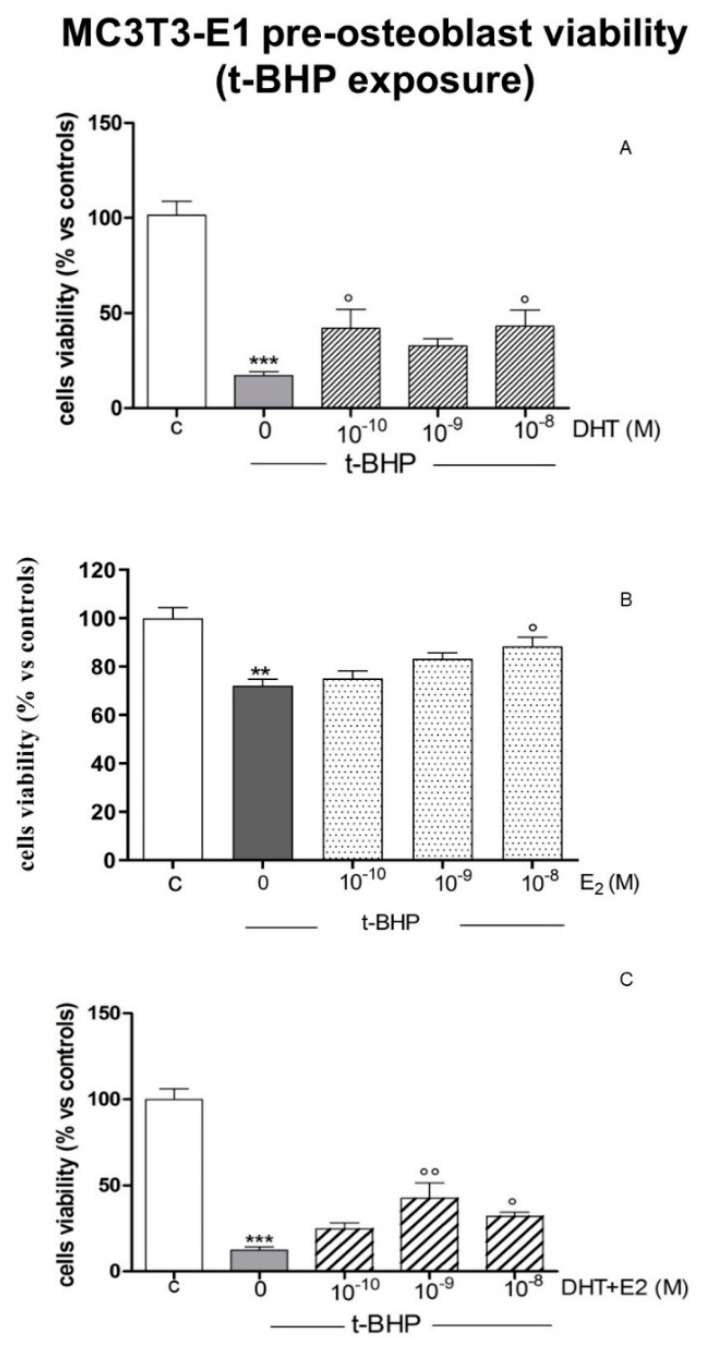
Effects of steroids on counteracting t-BHP-induced oxidative stress in pre-osteoblast MC3T3-E1 cells. Beneficial effects of steroids (10^−10^–10^−8^ M) on t-BHP-induced cytotoxicity in MC3T3-E1 cells. Cells were pre-treated with E2 or DHT, or a combination of both (doses between 10^−10^ M to 10^−9^ M), 24 h before being treated with t-BHP (250 µM for 3 h). Cell viability was measured by MTT assay 2 h after treatment. (**A**): effects of DHT on t-BHP-induced cytotoxicity in MC3T3-E1 cells; (**B**): effects of E2 on t-BHP-induced cytotoxicity in MC3T3-E1 cells; (**C**): effects of a co-treatment E2 + DHT (both at 10^−8^ M and 10^−9^ M) on t-BHP-induced cytotoxicity in MC3T3-E1 cells. Column C represents the control (w/o steroids treatment and w/o t-BHP exposure), column 0 represents the t-BHP group w/o steroids. Columns 10^−10^, 10^−9^, 10^−8^ represent the cells treated with increasing doses of steroids and exposed to t-BHP. Data are presented as the mean values ± SEM of six replicates within a single experiment. The experiment was repeated three times. Differences between groups were evaluated by one-way ANOVA, followed by Bonferroni post-hoc test. ** *p* < 0.01; *** *p* < 0.001 vs. control. ° *p* < 0.5; °° *p* < 0.01 vs. t-BHP.

We studied the ability of E2 and DHT to counteract the effects of t-BHP-induced oxidative stress on MC3T3E1 pre-osteoblast viability (Figure 1). The E2 and DHT exposure were performed at concentrations ranging from 10^−8^ M to 10^−10^ M, 24 h before treating t-BHP. Figure 1 shows that 3 h treatment with 250 μM t-BHP (0 column), selected based on previous studies [34], significantly reduced MC3T3-E1 cell viability compared to the control group (c column), as analyzed by the MTT test. Figure 1A shows the effects of DHT on t-BHP-induced cytotoxicity in pre-osteoblasts MC3T3-E1 cells; Figure 1B shows the effects of E2 on t-BHP-induced cytotoxicity in pre-osteoblasts MC3T3-E1 cells; panel C shows the effects of E2 + DHT on t-BHP-induced cytotoxicity in pre-osteoblasts MC3T3-E1 cells. Both DHT (Figure 1A) and E2 (Figure 1B) significantly reversed the detrimental action of t-BHP on cell viability, reaching a maximal protective activity at 10^−8^ M. Interestingly, the cotreatment with both steroids shows a protective effect from 10^−9^ M concentration (DHT + E2) (Figure 1C).

### 3.2. Effects of Steroids on Counteracting t-BHP-Induced Oxidative Stress in 7-Days Differentiated MC3T3-E1 Cells on Cells Viability

**Figure 2:**
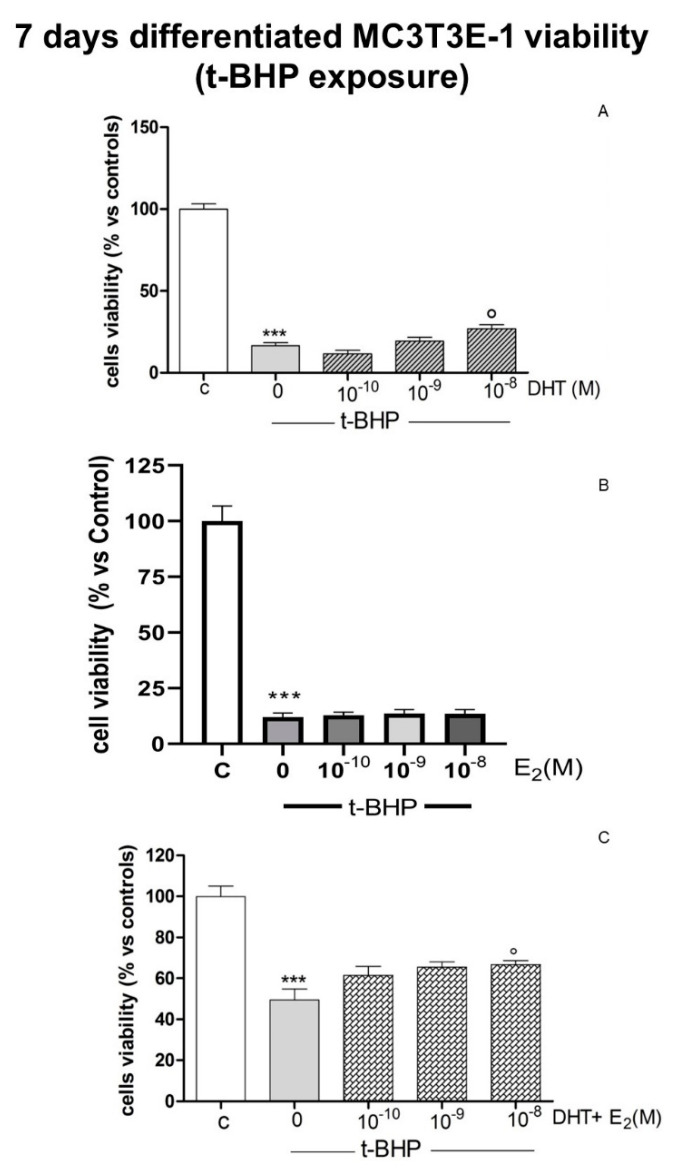
Effects of steroids on counteracting t-BHP-induced oxidative stress in 7-day differentiated osteoblasts MC3T3-E1 cells. Effects of steroids (10^−10^–10^−8^ M) on t-BHP-induced cytotoxicity in 7 day-differentiated osteoblasts MC3T3-E1 cells. Cells were pre-treated with E2 or DHT or a combination of both (doses between 10^−10^ M and 10^−9^ M), 24 h before being treated with t-BHP (125 µM for 3 h). We measured cell viability by MTT assay 2 h after treatment. (**A**): effects of DHT on t-BHP-induced cytotoxicity in 7-day-differentiated osteoblasts MC3T3-E1 cells; (**B**): effects of E2 on t-BHP-induced cytotoxicity in 7-day-differentiated osteoblasts MC3T3-E1 cells; (**C**): effects of a co-treatment of E2 + DHT on t-BHP-induced cytotoxicity in 7-day-differentiated osteoblasts MC3T3-E1 cells. We represented data as the mean ± SEM of six replicates within a single experiment. Column C represents the control (w/o steroids treatment and w/o t-BHP exposure), Column 0 represents the t-BHP group w/o steroids. MC3T3-E1 cells were treated via increasing concentrations of steroids 10^−10^, 10^−9^, and 10^−8^ M were exposed to t-BHP. The experiment was repeated three times. Differences between groups were evaluated by one-way ANOVA, followed by Bonferroni post-hoc test. *** *p* < 0.001 vs. control, ° *p* < 0.05 vs. t-BHP.

**Figure A1:**
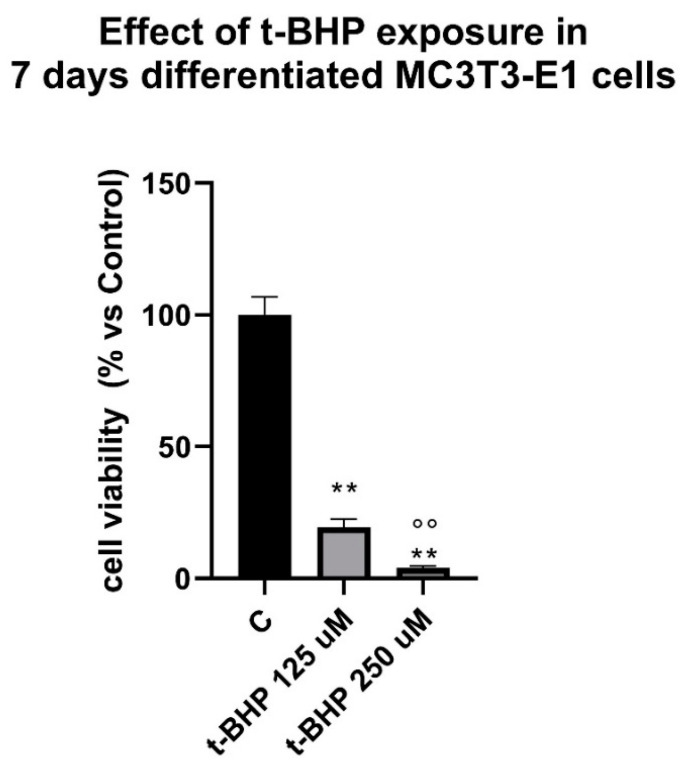
The figure shows the effects of t-BHP on 7-day-MC3T3E1-differentiated cells. We used 250 µM or 125 µM for 3 h of t-BHP to induce oxidative stress. The figure shows that 7-day-MC3T3E1-differentiated cells are more sensitive to t-BHP 250 µM than 125 µM. ** *p* < 0.001 vs. control. °° *p* < 0.05; vs. t-BHP 125 µM.

We then evaluated E2 and DHT’s ability to counteract t-BHP-induced oxidative stress in 7-days differentiated osteoblast MC3T3E1 cells (Figure 2). We used a 125 µM t-BHP for 3 h to induce oxidative stress, since we observed that 7-day differentiated MC3T3-E1 cells are more sensitive to oxidative stress (Figure A1, Appendix A section). The differentiation of MC3T3-E1 cells was induced by supplementation with L-ascorbic acid 50 μg/mL and β-glycerolphosphate, as previously described [40].

The E2 and DHT exposure was performed at concentrations ranging from 10^−8^ M to 10^−10^ M, including the cotreatment, 24 h before treating with t-BHP. As shown in Figure 2, only 10^−8^ M DHT (Figure 2A) displays a slightly, but significant counteracting effect on the adverse action of t-BHP on cell viability. E2 alone does not display any protective effect (Figure 2B), but A co-pretreatment (starting from 10^−9^ M) of E2 plus DHT (DHT + E2) (Figure 2C) partially revert the negative impact of t-BHP.

### 3.3. Effects of Steroids on Counteracting t-BHP-Induced Oxidative Stress in Osteocytes Like Cells, MLOY-4

**Figure 3:**
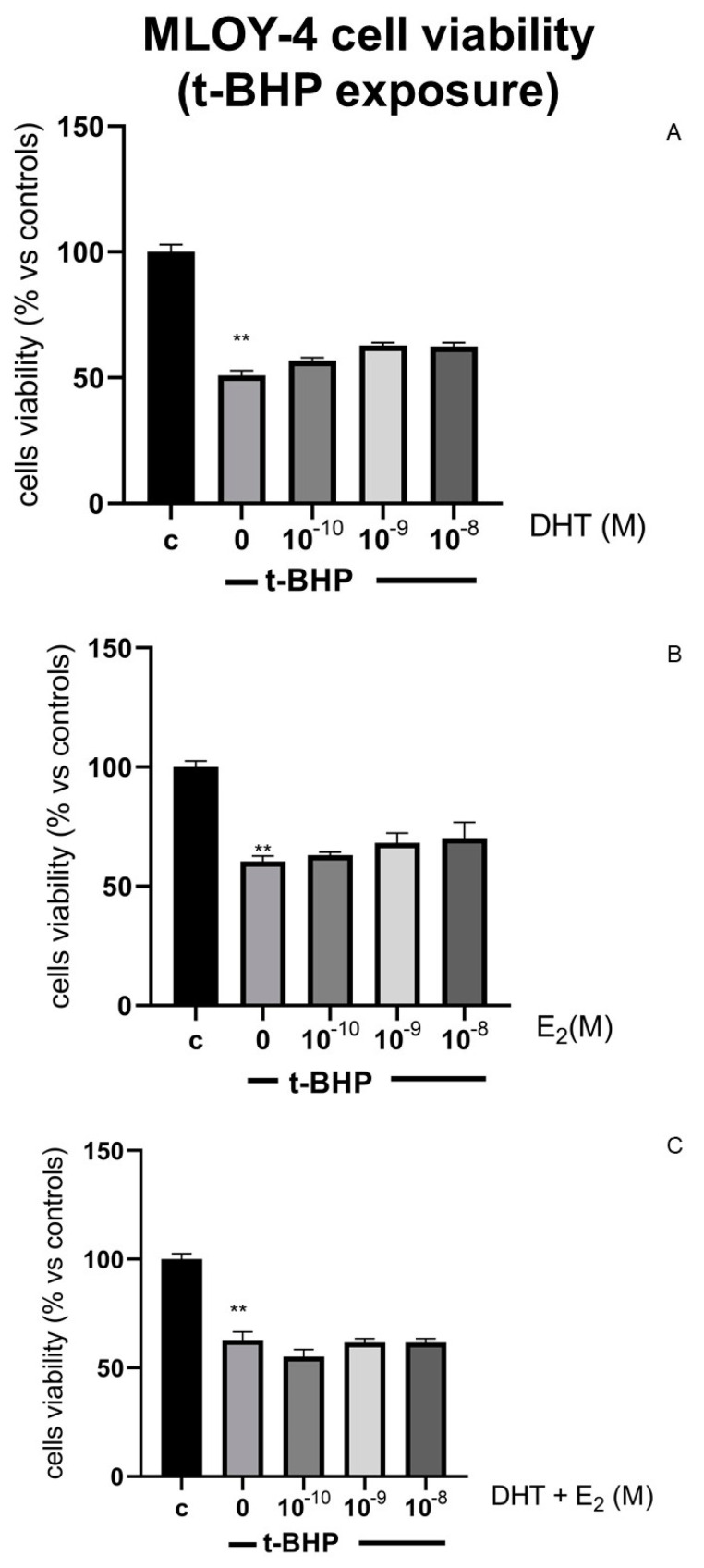
Effects of steroids on counteracting t-BHP-induced oxidative stress in osteocyte-like cells, MLOY-4. Effects of steroids on t-BHP-induced cytotoxicity in osteocyte-like cells, MLOY-4. Cells were pre-treated with E2 or DHT (range between 10^−10^ M to 10^−9^ M), 24 h before being treated with t-BHP (125 µM for 3 h). Cell viability was measured by MTT assay 2 h after treatment. (**A**) shows the effects of DHT on t-BHP-induced cytotoxicity in MLOY-4 cells; (**B**) shows the effects of E2 on t-BHP-induced cytotoxicity in MLOY-4 cells; (**C**) shows the effects of a co-treatment of E2 + DHT on t-BHP-induced cytotoxicity in MLOY-4 cells. Data are represented as the mean ± SEM of six replicates within a single experiment. Column C represents the control (w/o steroids treatment and w/o t-BHP exposure); column 0 represents the t-BHP group w/o steroids. The columns 10^−10^, 10^−9^, 10^−8^ represent the columns with increasing doses of steroids and exposed to t-BHP. Differences between groups were evaluated by one-way ANOVA, followed by Bonferroni post-hoc test. The experiment was repeated three times. ** p < 0.05 vs. t-BHP.

**Figure A2:**
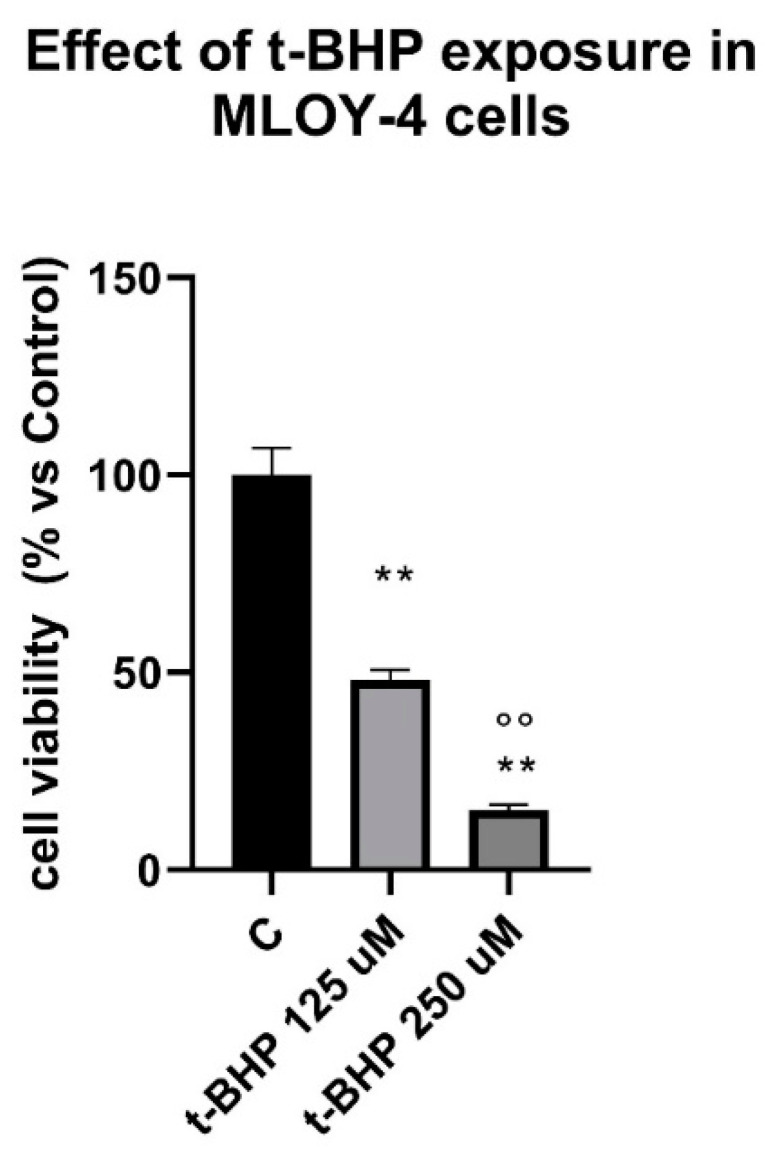
The figure shows the effects of t-BHP on osteocytes, MLOY-4. We used 250 µM or 125 µM for 3 h of t-BHP to induce oxidative stress. The figure shows that MLOY-4 cells are more sensitive to t-BHP 250 µM µM than 125 µM. ** *p* < 0.001 vs. control. °° *p* < 0.05; vs. t-BHP 125 µM.

We also analyzed the ability of E2 and DHT to counteract t-BHP induced oxidative stress in osteocyte-like cells, MLOY-4 (Figure 3). The E2 and DHT exposure was performed at concentrations ranging from 10^−8^ M to 10^−10^ M (Figure 3A,B), including the cotreatment (DHT + E2, Figure 3C), 24 h before treating t-BHP. The E2 and DHT exposure was performed at a concentration ranging from 10^−10^ to 10^−8^ M, 24 h before treating t-BHP. We used a 125 µM for 3 h of t-BHP to induce oxidative stress, since MLOY-4 cells are more sensitive to oxidative stress (Figure A2, Appendix A section). Neither DHT (Figure 3A,C) nor E2 (Figure 3B,C) reversed the adverse action of t-BHP on cell viability at all tested concentrations.

### 3.4. Effects of Steroids on Counteracting Dexamethasone-Induced Oxidative Stress in 7-Days Differentiated MC3T3-E1 Cells and in Osteocyte-Like Cells MLOY-4

**Figure 4:**
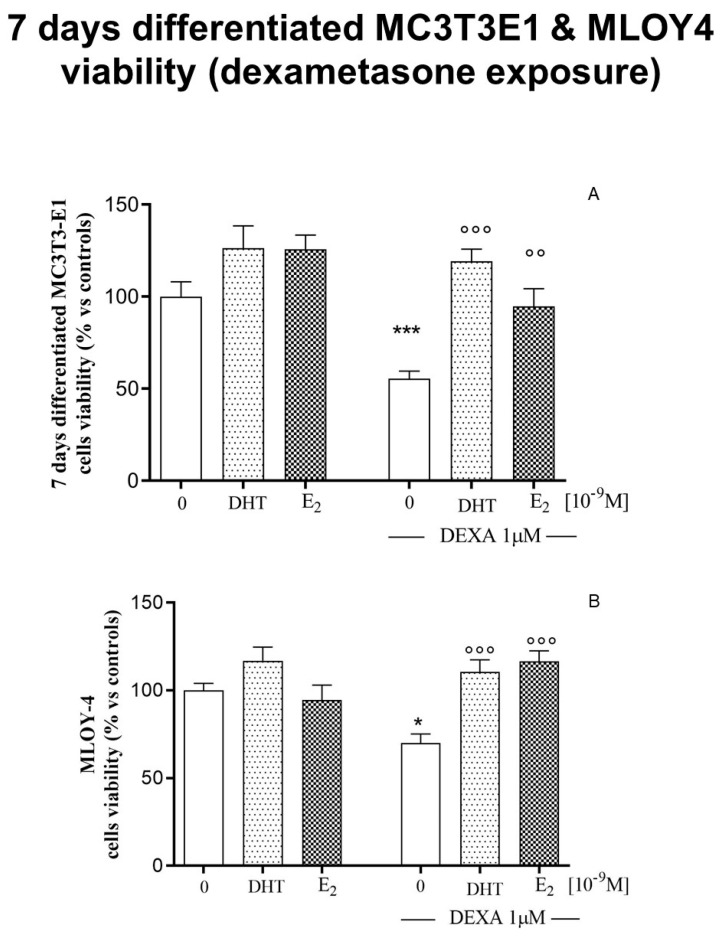
Effects of steroids on counteracting dexametasone-induced oxidative stress in osteocyte-like cells, MLOY-4 and in differentiated osteoblasts, MC3T3. Effects of 10^−9^ M steroids on dexametasone-induced cytotoxicity in osteocyte like cells, MLOY-4, and differentiated osteoblasts, MC3T3-E1. Cells were pre-treated with E2 or DHT, 24 h before being treated with dexametasone (1 µM for 48 h). We measured cell viability by MTT assay 2 h after treatment. (**A**): effects of steroids on dexamethasone-induced cytotoxicity in differentiated MC3TE1 cells; (**B**) shows the effects of steroids on dexamethasone-induced cytotoxicity in MLOY-4 cells. 0 column indicates the group without and with dexamethasone. Data are represented as the mean ± SEM of six replicates within a single experiment. The experiment was repeated three times. Differences between groups were evaluated by one-way ANOVA, followed by Bonferroni post-hoc test *** *p* < 0.001; * *p* < 0.05 vs. control without dexamethasone; °°° *p* < 0.01; °° *p* < 0.01 vs. control with dexamethasone.

Finally, we analyzed E2 and DHT’s ability to counteract dexamethasone-induced oxidative stress in both differentiated osteoblasts MC3T3E-1 and osteocyte-like cells MLOY-4 (Figure 4). The E2 and DHT exposure was performed at 10^−8^ M, 24 h before the exposure for 48 h to 1 µM dexamethasone. To verify the effects on recovering cell viability using a different source of ROS, we used the higher tested dose of steroids, which was shown to be effective (10^−8^ M). As shown in Figure 4, both DHT and E2 (Figure 4A) significantly reversed the adverse action of dexamethasone on cell viability, on both 7-day-differentiated MC3T3-E1 osteoblasts (Figure 4A) and MLOY-4 osteocytes (Figure 4B).

### 3.5. Transcriptomic Profile: Effect of Oxidative Stress and Steroids on Gene Expression

**Figure 5:**
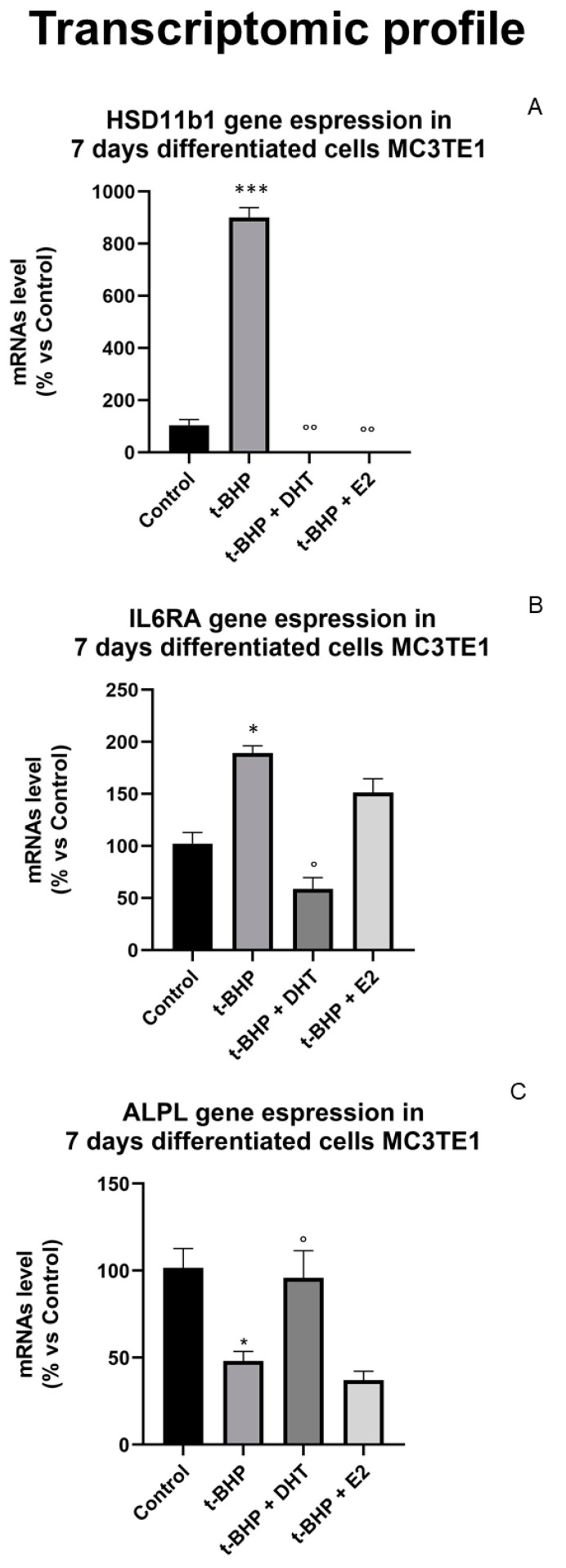
Effects of steroids on transcriptomic profile of genes related to osteoporosis pathway. Effects of steroids (10^−8^ M, 24 h before t-BHP) in differentiated MC3T3-E1 cells exposed to t-BHP (125 µM, 3 h) on gene expression of HSD11b1 (**A**), IL6RA (**B**), ALP (**C**). The figure shows the effects of steroids in counteracting the gene expression disruption induced by t-BHP exposure. Data are represented as the mean ± SEM of six replicates within a single experiment. Differences between groups were evaluated by one-way ANOVA, followed by Bonferroni post-hoc test *** *p* < 0.001; * *p* < 0.05 vs. control. ° *p* < 0.05, °° *p* < 0.01; vs. t-BHP.

To understand the molecular mechanisms underlying the outcome of oxidative stress-induced aging in MC3T3 cells and the ability of DHT e E2 to revert it, we performed a transcriptomic analysis using a PCR array. Specifically, we focused on the profile of genes involved in osteoporosis due to their relevance in aging (Table 1 and Figure 5). MC3T3-E1 cells were exposed to t-BHP (125 µM, 3 h). The E2 and DHT exposure (10^−8^ M) was performed 24 h before treating t-BHP.

t-BHP exposure affected several genes, mainly alkaline phosphatase (*ALPL*), involved in bone mineralization); calcitonin receptor (*CALCR*), involved in osteoporosis development; chloride voltage-gated channel 7 (*CLCN7*), involved in bone diseases such as osteopetrosis; hydroxysteroid 11-β dehydrogenase 1 (*HSD11B1*), involved in the conversion of cortisol to the inactive metabolite cortisone; interleukin-6 (*IL6*); interleukin-6 receptor subunit α (*IL6RA*); methylenetetrahydrofolate reductase (*MTHFR*); nuclear factor of activated t-cells 1 (*NFATC1*), a nuclear transcription factor involved in the control of cytokine gene expression; runt-related transcription factor 2 (*RUNX2*), involved in the regulation of the transcription of genes related to osteoblastogenesis; sex-hormone-binding flobulin precursor (*SHBG*), *TNFSR11B*, also known as osteoprotegerin, involved in the inhibition of osteoclastogenesis; and vascular endothelial growth factor (*VEGFa*) (see Table 1 and Figure 5), probably through the impairment of the epigenetic mechanism. We also found that treatment with steroids counteracts the alteration in the gene expression profile in a dimorphic way for *ALPL*, *HSD11b1*, and *IL6RA* (see Table 1 and Figure 5). DHT reverts the inhibitory effect on ALPL gene expression and reduces the gene expression of IL6RA. Both E2 and DHT revert the impact of t-BHP on HSD11b1 and SHBG gene induction.

### 3.6. Epigenetic Mechanism: Effect of Oxidative Stress and Steroids on Histone Modification

**Figure 6:**
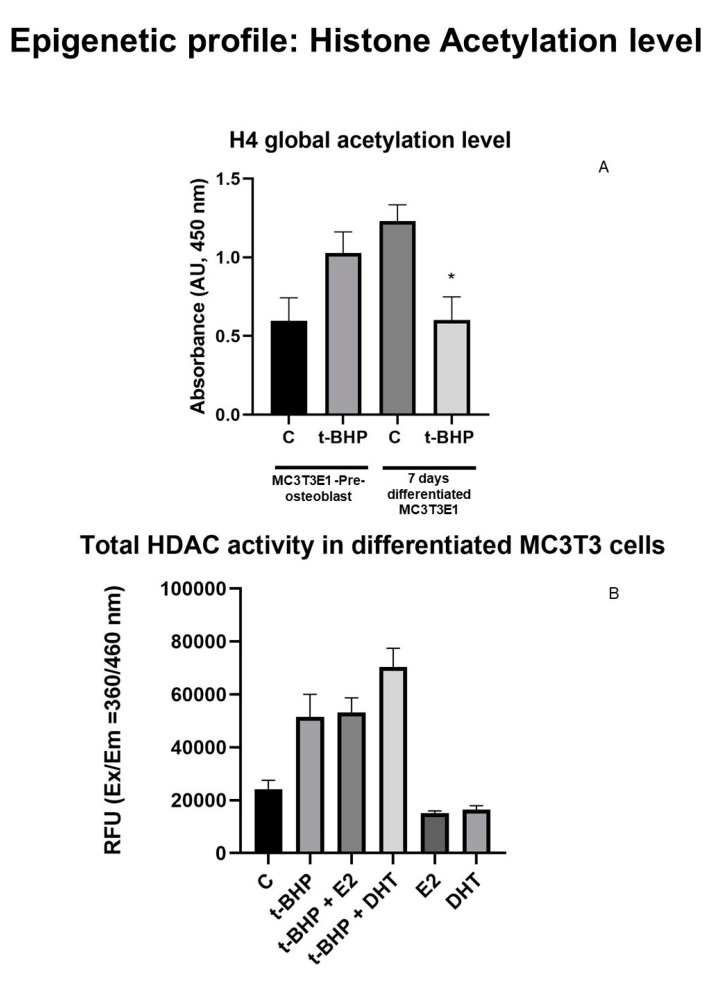
Effects of oxidative stress and steroids on histone acetylation. Effects of exposure to t-BHP (250 µM in pre-osteoblastic cells and 125 µM in 7 days differentiated cells) and steroids (10^−9^ M) on histone acetylation. (**A**): effect of t-BHP on H4 global acetylation level in pre-osteoblasts and differentiated osteoblasts. t-BHP reduces the H4 acetylation level only in differentiated osteoblasts. * *p* < 0.05 vs. control. (**B**): effects of steroids (10^−9^ M) in differentiated osteoblasts MC3T3-E1 cells exposed to t-BHP (125 µM, 3 h) on total HDAC enzymatic activity. According to previous data, t-BHP induces the enzymatic activity of HDAC. Steroids treatment does not restore HDAC activity to the control level. Data are represented as the mean ± SEM of six replicates within a single experiment. Differences between groups were evaluated by one-way ANOVA followed by Bonferroni post-hoc test. The experiment was repeated twice. * *p* < 0.05 vs. control.

As cited above, bone cell activities are also regulated by epigenetic mechanisms. For this purpose, we investigated the possible epigenetic mechanisms by which sex steroids counteract the disruptive effects on osteoepigenome induced by oxidative-stress-mediated aging (Figure 6). As shown in the Figure 6A, a 3 h treatment with t-BHP (250 µM in pre-osteoblastic cells and 125 µM in 7-day-differentiated cells) reduces the global contents of H4 acetylation in 7-day-differentiated osteoblasts but not in pre-osteoblasts. Therefore, since HDACs regulate the dynamic balance between acetylation and deacetylation of histones, we have further evaluated the histone deacetylase total activity (HDACs I and II classes) in 7-day-differentiated cells. As shown in Figure 6B, we found that 7-days differentiated MC3T3-E1 cells show a significant increase in the histone deacetylase total activity (HDAC) after t-BHP exposure (125 µM, 3 h). Treatment with hormonal steroids (10^−8^ M, 24 h before treating t-BHP) does not restore the physiological level of the histone deacetylase enzymatic activity.

**Figure 7:**
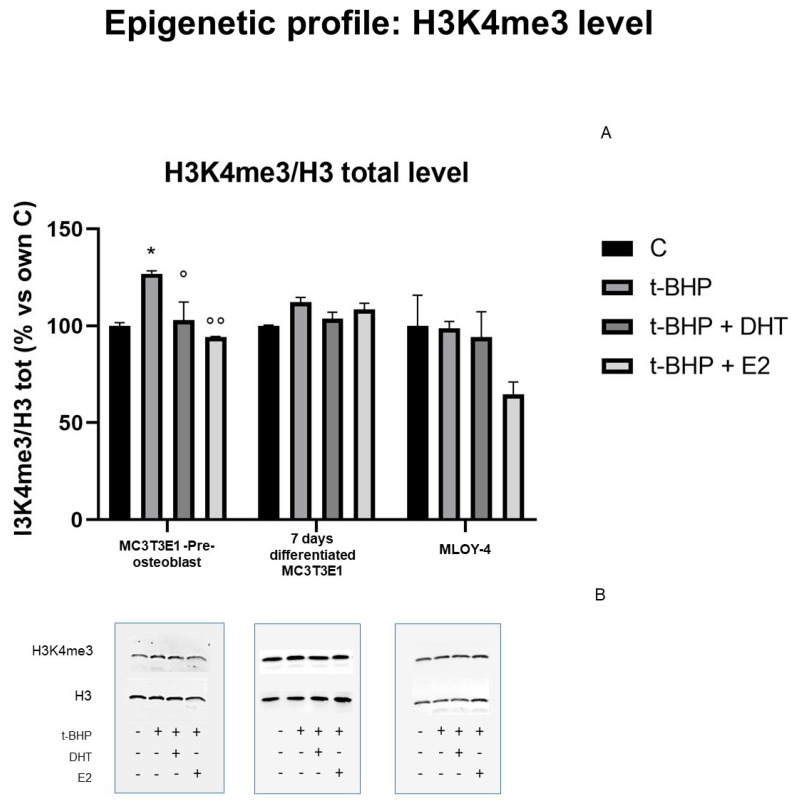
Effects of oxidative stress and steroids on H3K4me3. Effects of exposure to t-BHP (250 µM in pre-osteoblastic cells, 125 µM in 7-day-differentiated cells and osteocyte-like cells, MLOY4) and steroids (10^−9^ M) on H3K4me3. (**A**): densitometric analysis from Western blot analysis for H3K4me4. The signal from H3K4me3 was normalized against the total histone H3. t-BHP exposure only induces H3K4me3 in pre-osteoblasts and steroids treatment (more consistent for E2) restores the basal condition. (**B**) Representative Western blot for H3K4me3 and total histone H3. Data are represented as the mean ± SEM of three replicates within a single experiment. The experiment was repeated twice. Differences between groups were evaluated by one-way ANOVA, followed by Bonferroni post-hoc test. * *p* < 0.05 vs. control, °° *p* < 0.01 vs. t-BHP, ° *p* < 0.05 vs. t-BHP.

We did not observe any beneficial effects of steroids on global acetylation levels. From our previous work, we hypothesized that steroids could interplay with histone demethylases [12]. Therefore, we investigated the effects of oxidative stress t-BHP-induced and steroids on trimethylation of lysine 4 on histone H3 (H3K4me3) global level on both pre-osteoblasts, 7-day-differentiated osteoblasts and osteocytes (Figure 7). As shown in Figure 7A,B from densitometric analysis on Western blotting experiments, we found that t-BHP only increases H3K4me3 levels in pre-osteoblasts, which is related to active transcription [30]; both E2 and DHT treatment restore H3K4me3′s physiological levels.

### 3.7. Effects of Hormonal Steroids on MC3T3E-1 Cell Migration

As underlined in our previous paper [41], analysis of the migratory potential of osteoblastic cells could be considered as a new parameter for an innovative therapeutical strategy for bone disease.

**Figure 8:**
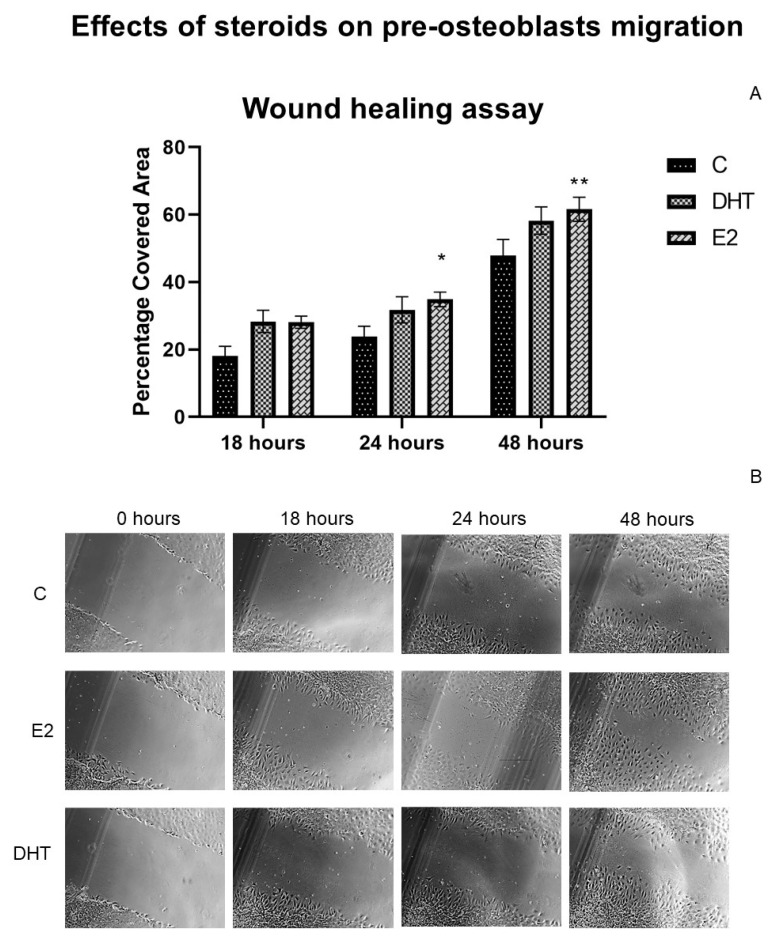
Effects of steroids’ milieu on pre-osteoblast migration. E2 promotes the migration of MC3T3-E1 cells in a wound-healing assay. Steroid treatment (10^−9^ M, DHT, and E2) was performed 24 h before the scratch. (**A**) quantification of wound closure. The graph represents the wound width as the percentage of the closure of the original wound. The area of the wound size was detected using ImageJ Software. Data are presented as the mean ± SEM of eight replicates within a single experiment. The experiment was repeated twice. Differences between groups were evaluated by two-way ANOVA, followed by Bonferroni post-hoc test * *p* < 0.05 ** *p* < 0.01 vs. control. (**B**) pictures of representative microscope fields of wound closure using Olympus U-CMAD3 phase-contrast microscope equipped with a Zeiss Axiocam ICc1 camera at 4x magnification. E2 action is evident in the promotion of osteoblast migration.

We studied the effects of steroids on bidimensional cell migration (or cell motility), performing a scratch assay (Figure 8). MC3T3-E1 cells were exposed to steroids (10^−9^ M concentration) for 24 h before and immediately after the scratch; then, cells were observed at 18, 24, and 48 h. As shown in Figure 8A, MC3T3-E1 cells treated with E2 exhibited a faster wound-healing rate than control untreated cells at 18, 24, and 48 h, reaching statistical significance at 24 h.

### 3.8. β-Catenin Is Involved in E2 Effect on Cell Motility

**Figure 9:**
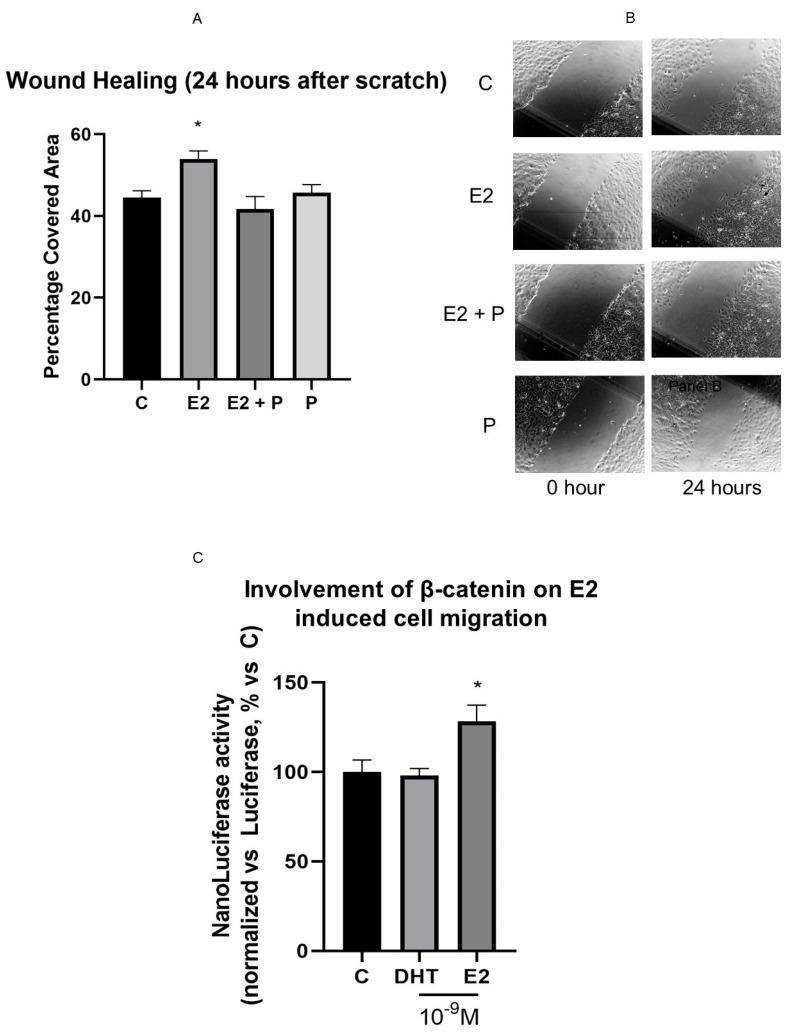
Involvement of β-catenin pathway in steroid-promoted migration. Panel (**A**): Procaine, a Wnt/β-catenin signaling pathway inhibitor, removed the wound-healing effects of E2 (10^−9^ M) in MC3T3-E1 cells. Cells were incubated with procaine (2 µM) 30 min before E2. (**A**): quantification of wound closure. The graph represents the wound width as the percentage of the closure of the original wound. The area of the wound size was detected using ImageJ Software. (**B**): pictures of representative microscope fields of wound closure. The involvement of the β-catenin pathway was evident in steroid-promoted migration. The data are presented as the mean ± SEM of eight replicates within a single experiment. The experiment was repeated twice. * *p* < 0.05 vs. control. (**C**): role of E2 in inducing β-catenin transcriptional activity. MC3T3-E1 cells were transiently co-transfected with pGL4.54 (luc2TK) pNL (NLucP/TCF-LEF-RE) and treated for 48 h with steroids (10^−9^ M). The level of β-catenin promoter transactivation is expressed as a percentage, i.e., ratio between nanoluciferase and firefly luciferase activity. We represented the data as the mean ± SEM of eight replicates. Differences between groups were evaluated by one-way ANOVA, followed by Bonferroni post-hoc test * *p* < 0.05 vs. control.

According to our previous studies, the β-catenin pathway is involved in controlling bone remodeling and osteoblast migration; therefore, we investigated its involvement in cell migration (Figure 9) [41] First, we tested the involvement of β-catenin in the enhancing E2 effect on MC3T3-E1 cell migration using procaine (P), an inhibitor of the Wnt [44,45]. As shown in Figure 9A,B, pre-treatment with 2 µM P per se did not modify the MC3T3-E1 cell’s ability to cover the wound but completely prevented the positive action of E2 on MC3T3-E1 cell motility.

We also confirmed the involvement of the β-catenin pathway by analyzing the effect of sex steroids on β-catenin transcriptional activity in MC3T3-E1 cells. For this purpose, we used a gene reporter assay. We used two different plasmids: one containing a nanoluciferase gene under the control of the TCF/LEF responsive element, responsive to β-catenin activation, the other codifying for the firefly luciferase gene under the control of a strong, constitutive promoter. Both plasmids were commercially available from Promega. As reported in Figure 9C, only E2 induced β-catenin transcriptional activity, as shown by a statistically significant increase in luciferase activity compared to untreated MC3T3-E1 cells.

## 4. Discussion

Correct bone mass acquisition and maintenance involve consecutive osteoblast migration, differentiation, and mineralization [46]. An appropriate hormonal milieu is necessary to maintain the proper bone mass. A reduction in sex steroid level causes a loss of bone mass and strength [1]. This alteration, coupled with increased oxidative stress, contributes to the development of osteoporosis, one of the most common metabolic aging disorders [1]. Almeida and coworkers [26] reported the detrimental effects of the loss of steroids, and the oxidative stress related to aging, on bone stress in C57BL/6 mice 4–31 months old. In this paper, it was concluded that oxidative stress may be a critical pathogenetic mechanism of age-related bone alteration, and that a loss of estrogens or androgens could accelerate the effects of aging on bone by decreasing defense against oxidative stress [26].

Hendrickx and colleagues [47] underline that aging represents a nonmodifiable risk factor for osteoporosis, but lifestyle (nutrition, exercise, increase in oxygen reactive species, hormonal milieu) could influence bone structure. [47]. Epigenome also contributes to the susceptibility to developing osteoporosis. Identifying these risk factors has already partially unraveled therapeutic targets for osteoporosis, evidencing the importance of basic research into the pathogenetic mechanisms of osteoporosis [47].

**Scheme 1:**
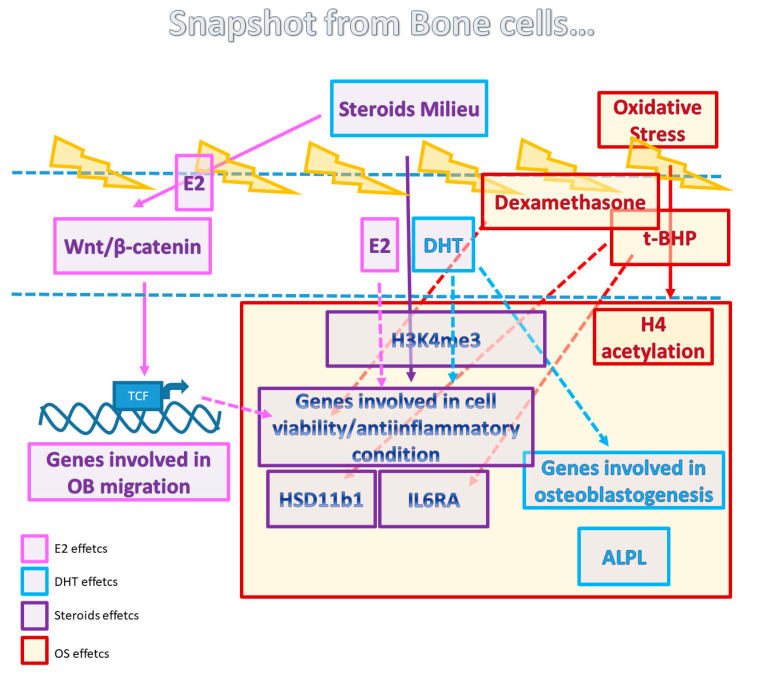
A snapshot from bone cells. The scheme shows a possible strategy used by steroids in counteracting oxidative stress. The violet box (and arrows) indicates the effects of both DHT and E2. The pink box (and arrows) shows the E2 effects. The blue box (and arrows) indicates the DHT effects. The red box (and arrows) shows the effects of oxidative stress.

However, the interplay between sex steroids and oxidative stress remains to be fully elucidated, as well as the regulative role of epigenetic mechanisms. The present study demonstrates that steroids have a dimorphic effect on cell viability and motility in different bone cell types, possibly following different mechanisms. DHT shows higher effects on recovery viability for pre-osteoblast, 7-days differentiated osteoblasts, and osteocytes after the induction of oxidative stress. DHT shows a powerful effect compared to E2 on cell viability, even if the E2 impact is more evident in the induction of motility/migration. From the literature, the anabolic role of DHT is entirely known, whereas the role of E2 is more focused on the control of bone remodeling. We also found that t-BHP can disrupt the transcriptomic profile of a panel of genes involved in a correct bone mass acquisition and related to inflammation. The HSD11b1 gene is related to glucocorticoids metabolism [48]. The induction of this gene could be involved in a cell’s attempt to respond to oxidative stress. It is possible that the DHT and E2 protective action observed in 7-day-differentiated osteoblasts and osteocytes exposed to dexamethasone could be related to the induction of HSD11b1 gene expression caused by dexamethasone, as was found by Kaur in primary human osteoblasts and MG-63 osteosarcoma cells [49]. According to our results, the literature shows that steroids, especially E2, could exert an inhibitory effect on HSD11b1 upregulation, which could be related to an inflammatory state [48,49]. IL6RA is related to the inflammation pathway, whether IL6RA could act as a positive regulator of osteoblasts [50]. Moreover, oxidative stress disrupts the correct timing of osteoblastogenesis (affecting ALPL and RANKL) and ROS affects osteoblastogenesis, shifting the balance of mesenchymal stem cells towards the adipocyte phenotype [2]. DHT counteracts the action of t-BHP on bone metabolism (see ALPL gene expression), but not E2. The genomic action of DHT seems to play a role in protecting osteoblastogenesis from the alterations induced by oxidative stress. According to the literature, DHT plays an anabolic role in bone [51]. Together (see also Table 1), these data allow for the hypothesis that E2 and DHT use different epigenetic/transcriptomic strategies to counteract the genomic/epigenomic action of t-BHP (see Scheme 1).

ROS cause a spectrum of responses, ranging from proliferation to growth or differentiation arrest, or to cell death, by activating numerous signaling pathways, heat shock factor (HSF), and MAPKs, including extracellular signal-regulated kinases (ERKs), c-Jun-N terminal kinase (JNK), p38 MAPK and ERK5 [52]. The chromatin modeling, mediated by t-BHP, induces changes in the transcriptomic profile; different pathways can cause changes in chromatin conformation [30]. We found that steroids could partially revert this effect using an epigenetic mechanism. Indeed, both DHT and E2 could partially counteract the transcriptomic change and epigenomic alteration, restoring the H3K4me3 physiological level. Moreover, E2 could also use another transcriptional pathway, using β-catenin signaling, which is involved in controlling the directional migration capacity of osteoblast-like cells in physiological conditions, as previously demonstrated [41].

It is well documented that the Wnt/β-catenin signaling pathway is an essential regulator of cell–cell adhesion and cell migration [53,54]. Canonical Wnt signaling is mediated by a multi-protein complex, including glycogen synthase kinase-3 β (GSK-3β). The transcriptional regulator β-catenin is central to the canonical Wnt signaling. In the absence of Wnt ligands, β-catenin is targeted for proteasomal degradation [55].

Pre-treatment with procaine, a local anesthetic drug known to inhibit the Wnt/β-catenin pathway [45,46,56], prevents the increase in wound-healing activity induced by E2. A similar effect using a nutraceutical compound was reported from our recent paper [41]. As a confirmation that the canonical Wnt signaling pathway is involved in the effect of E2 on MC3T3-E1 cell migration, we found that steroid treatment increased β-catenin transcriptional activity.

Recent evidence indicates that the bone epigenome exerts a fundamental role in the mechanisms impacting bone cell activities [57]. Osteoblast’s epigenetic regulation may affect gene expression through post-translational histone modifications, miRNA-mediated post-transcriptional regulation, and DNA methylation [28,56]. In the present study, we provide new data on the possible effects of oxidative stress on epigenetic mechanisms and on the counterregulatory effects of steroids on MC3T3-E1 cells and MLOY4.

t-BHP shows a different behavior on epigenetic hallmarks, as seen in the function of the differentiation degree of the cells. In pre-osteoblasts, t-BHP increases the total acetylation level of H4 and does not significantly affect HDAC activity. On the contrary, in 7-day-differentiated cells, t-BHP induces HDAC total activity and reduces the global level of H4. It is known that acetylation neutralizes the positive charge of histones, and thereby prevents the compaction of chromatin [58]. Interestingly, the negative impact of histone deacetylation on osteogenesis has been previously reported [59,60]. Moreover, we found that sex steroids do not restore the physiological level of global H4 acetylation [61]. This paper shows that oxidative stress disrupts epigenetic hallmarks, such as HDAC and HAT enzymatic activity and that E2 does not counteract this change.

The oxidative stress induced by t-BHP shows different effects if we consider another epigenetic mark. We investigated the impact of oxidative stress and the hormonal milieu on H3K4 trimethylation level, a significant histone modification related to transcription. Our previous studies have shown that H3K4me3 is sensitive to environmental exposure and that the H3K4me3 level is related to the steroid milieu [12,43]. t-BHP does not affect the H3K4me3 level in differentiated osteoblasts and osteocyte-like cells; on the contrary, in pre-osteoblasts, t-BHP induces H3K4me3, and both DHT and E2 counteract the negative effect of t-BHP on H3K4me3. The literature shows that H3K4me3, H3K27me2/3, H3K79me2/3, and H3K9me2/3 residues are engaged in a cellular reprogramming that drives gene expression in osteogenic differentiation [62]. The t-BHP induced deregulation could affect the correct timing of osteogenic differentiation, anticipating the proper maturation of osteoblasts. Our results are coherent with these data and underline the role of steroids in counteracting t-BHP disruption at the epigenetic and genomic levels.

Epigenetic mechanisms represent an important issue associated with normal and aberrant bone remodeling. However, this in vitro study shows that the hormonal milieu positive effect is not completely mediated by epigenome regulation, and acetylation is not involved in steroids’ action. A different perspective is provided by H3K4me3, which, as demonstrated in our previous studies, could represent a link between steroids and epigenome in responding to a negative cue.

## 5. Conclusions

Our results are summarized in the model described in Scheme 1 (Figure, Table and Scheme section). Steroids counteract the oxidative stress induced by t-BHP or dexamethasone with partially overlapping strategies: altering Wnt B-catenin and/or inflammatory pathways through epigenetic mechanisms. Our results are consistent with the working hypothesis that underlines the dimorphic action of steroids on disruptive effects of oxidative stress in bone cell regulation, which recapitulates the bone alteration observed in aging. The mechanism involved is highly complex and could involve both genomic and not-genomic pathways, together with epigenetic regulation.

Bone remodeling remains a highly complex, fine-tuned mechanism. Our studies insert a little piece of evidence in the sex-steroid effect seen in bones under adverse conditions, increasing the susceptibility to osteoporosis.

## Figures and Tables

**Table 1:** Transcriptomic profile affected by t-BHP exposure. Effects of t-BHP in gene expression of 7-day-differentiated cells. The analysis is focused on osteoporosis pathways. The effects of E2 and DHT pre-treatment are also shown. The red arrows indicate the t-BHP effect, the green arrows indicate the recovery of gene expression induced by DHT or E2, C indicates the control group.

Gene	Acronym	t-BHP Effect	DHT + t-BHP Effect	E2 + t-BHP Effect
Alkaline Phosphatase	ALPL	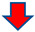	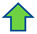 = C	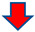
Calcitonin Receptor	CALCR	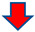	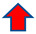	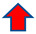
Chloride Voltage Gated Channel 7	CLCN7	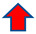	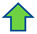	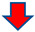
Hydroxysteroid 11-β Dehydrogenase 1	HSD11B1	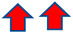	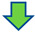 = C	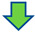 = C
Interleukin-6	IL6	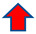	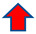	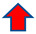
Interleukin-6 receptor subunit alpha	IL6-RA	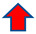	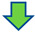 = C	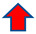
Methylenetetrahydrofolate Reductase	MTHFR	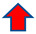	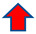	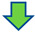
Nuclear Factor Of Activated T Cells 1	NFATC1	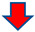	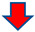	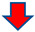
Runt-related transcription factor 2	RUNX-2	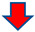	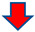	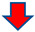
Sex hormone-binding globulin precursor	SHBG	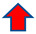	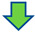 = C	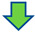 = C
Osteoprotegerin	TNFSR11B1	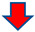	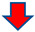	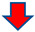
Vascular endothelial growth factor	VEGFA	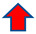	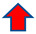	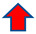

## Data Availability

Results and data can be found at the University of Milan.
